# Airway immune response in the mouse models of obesity-related asthma

**DOI:** 10.3389/fphys.2022.909209

**Published:** 2022-08-16

**Authors:** Jingwei Kong, Fan Yang, Minghua Bai, Yuhan Zong, Zhuqing Li, Xianghe Meng, Xiaoshan Zhao, Ji Wang

**Affiliations:** ^1^ School of Traditional Chinese Medicine, Beijing University of Chinese Medicine, Beijing, China; ^2^ National Institute of TCM Constitution and Preventive Medicine, Beijing University of Chinese Medicine, Beijing, China; ^3^ School of Chinese Medicine, Southern Medical University, Guangzhou, China

**Keywords:** obesity, asthma, obesity-related asthma, immune response, systemic inflammation

## Abstract

The prevalence rates of obesity and its complications have increased dramatically worldwide. Obesity can lead to low-grade chronic systemic inflammation, which predisposes individuals to an increased risk of morbidity and mortality. Although obesity has received considerable interest in recent years, the essential role of obesity in asthma development has not been explored. Asthma is a common chronic inflammatory airway disease caused by various environmental allergens. Obesity is a critical risk factor for asthma exacerbation due to systemic inflammation, and obesity-related asthma is listed as an asthma phenotype. A suitable model can contribute to the understanding of the in-depth mechanisms of obese asthma. However, stable models for simulating clinical phenotypes and the impact of modeling on immune response vary across studies. Given that inflammation is one of the central mechanisms in asthma pathogenesis, this review will discuss immune responses in the airways of obese asthmatic mice on the basis of diverse modeling protocols.

## Introduction

The worldwide prevalence of overweight and obesity has increased dramatically, posing a severe threat to human health from childhood to adulthood (Blüher, 2019; Chooi et al., 2019; Jaacks et al., 2019). According to the World Health Organization, obesity is defined as a body mass index (BMI) of over 30 kg/m^2^, whereas overweight is defined as a BMI of over 25 kg/m^2^ (World Health Organization, 2021). Regarding immune response, obesity can cause chronic low-grade systemic inflammation following excessive energy intake, adipose tissue (AT) expansion, and metabolic dysfunction (Ouchi et al., 2011; Bantulà et al., 2021; Rohm et al., 2022). An inflammatory state disrupts immune response in multiple tissues, leading to complications associated with increased disability and reduced quality of life, such as cardiovascular diseases and diabetes (Saltiel and Olefsky, 2017; Piché et al., 2020). Moreover, increased body mass increases individual susceptibility to airway hyper-responsiveness (AHR) and asthma development (Peters et al., 2018; Miethe et al., 2020). Asthma is a chronic inflammatory airway disease driven by various immune cells, cytokines, and other molecules (Kuruvilla et al., 2019; Global Initiative for Asthma, 2022; Hammad and Lambrecht, 2021). Obese asthmatics often suffer from frequent exacerbations, uncontrolled symptoms, impaired lung function, and limited therapeutic effects (Shore, 2008; Global Initiative for Asthma, 2022).

Given that asthma is a heterogeneous disease closely related to the immune system, underlying inflammatory mechanism is the primary target for research and treatment. The etiology remains unclear because of the complex interactions between asthma and obesity. Insights into the immune response of obesity-related asthma are essential to the clarification of phenotype definition and identification of novel prevention or treatment strategies. A suitable animal model is a prerequisite for exploring the underlying pathogenesis of this disease. However, the modeling process and airway immune responses differ across studies. The obese asthma model is a combination of obesity status (diet or genetic defects) and asthma induction (allergen selection and treatment). Mice are the most frequently used experimental animals in this model. To prevent the effects of experimental implementation on results, suitable and stable models of obesity-related asthma should be explored. This review summarizes modeling protocols for obese asthmatic mice and discusses the effects of modeling factors on different airway immune responses.

## Primary pathogenesis of asthma

Asthma is a common airway disease characterized by shortness of breath, chest tightness, and cough symptoms (Global Initiative for Asthma, 2022). Pathogens, such as external allergens, air pollutants, and viruses, invade a host’s respiratory tract and disrupt airway homeostasis (Lambrecht et al., 2019). Activated immune cells and infiltrating inflammatory molecules can mediate significant histological changes in asthma, such as airway obstruction, narrowing, and remodeling (Lambrecht et al., 2019; Hammad and Lambrecht, 2021). At the same time, it is imperative to note that the dominant immune cells differ between different asthma phenotypes and endotypes (Boonpiyathad et al., 2019; Agache et al., 2021).

### Immunological basis of asthma

Asthma is a heterogeneous disease with complex pathophysiological mechanisms (endotypes) and different clinical presentations (phenotypes) (Bantulà et al., 2021). Airway inflammation is characterized by the recruitment of effector immune cells, such as eosinophils, neutrophils, mast cells, innate lymphoid cells (ILCs), dendritic cells, T lymphocytes, and B lymphocytes (Lambrecht et al., 2019; Hammad and Lambrecht, 2021). These predominant cells can be divided into various subsets according to their responses to endogenous or exogenous stimulation. For example, macrophages can be divided into “classically activated” M1 induced by IFN-γ or lipopolysaccharide (LPS) and “alternatively activated” M2 macrophages triggered by IL-4 and IL-13 (Locati et al., 2020). Moreover, there are three main subsets of ILCs and CD4^+^ helper T (Th) cells according to their immune function, ILC1s, ILC2s, ILC3s, and their mirror counterparts Th1, Th2, and Th17 cells (Colonna, 2018).

Based on different immunological pathways, asthma could be classified into two endotypes—type 2-high (T2-high) and type 2-low (T2-low) (Hammad and Lambrecht, 2021). Type 2-high asthma endotype is featured by high levels of eosinophils, type 2 cytokines, and antigen-specific IgE antibodies in the sputum and/or blood (Fahy, 2015). ILC2s and Th2 cells play central roles in T2-high airway inflammation for innate and adaptive immunity, respectively. Various allergens can stimulate airway epithelial cells to secrete interleukin-33 (IL-33), IL-25, and thymic stromal lymphopoietin (TSLP) to activate ILC2s, which amplify airway inflammation by promoting the antigen presentation of dendritic cells and polarization of T cells (Hammad and Lambrecht, 2021). Large amounts of cytokines IL-4, IL-5, and IL-13 are further released and facilitate the synthesis of allergen-specific IgE and the accumulation of inflammatory cells, such as eosinophils, mast cells, basophils, and M2 macrophages (Lambrecht et al., 2019). The degranulation of mast cells and basophils releases various cytokines, chemokines, vasoactive amines, and lipid mediators. Consequently, this excessive type 2 immune response leads to increased mucus secretion and further tissue damage (Fahy, 2015).

Type 2-low asthma has attracted substantial interest because studies have revealed that only half of asthmatic patients suffer type 2 immunity-skewed airway inflammation (Hammad and Lambrecht, 2021). Compared with T2-high asthma, T2-low asthma has distinctive features, such as airway neutrophilia, corticosteroid resistance, Th1/Th17 polarization, and absence of type 2 immune production (Sze et al., 2020). Neutrophils are essential for the body’s immune surveillance and host defense due to their early recruitment and ability to eliminate invading pathogens efficiently. Overexuberant or persistent neutrophilic inflammation is implicated in the pathology of T2-low asthma (Patel et al., 2019). Moreover, Th1 cells, Th17 cells, ILC1s, ILC3s, and M1 macrophages are involved in this endotype, and their production IL‐1β, IL‐6, IL‐17, IFN‐γ, and TNF‐α are responsible for the recruitment and activation of inflammatory cells during asthma attacks. Remarkably, the most consistent pathways in T2-low asthmatics are those related to the inflammasome and IL-1β pathway (Hammad and Lambrecht, 2021). The activation of the nucleotide-binding oligomerization domain (NOD) -like receptor family pyrin domain-containing protein 3 (NLRP3) inflammasome and caspase-1 and elevated IL-6 and IL-1β levels have been implicated in the pathogenesis of this endotype (Kim et al., 2017).

**FIGURE 1:**
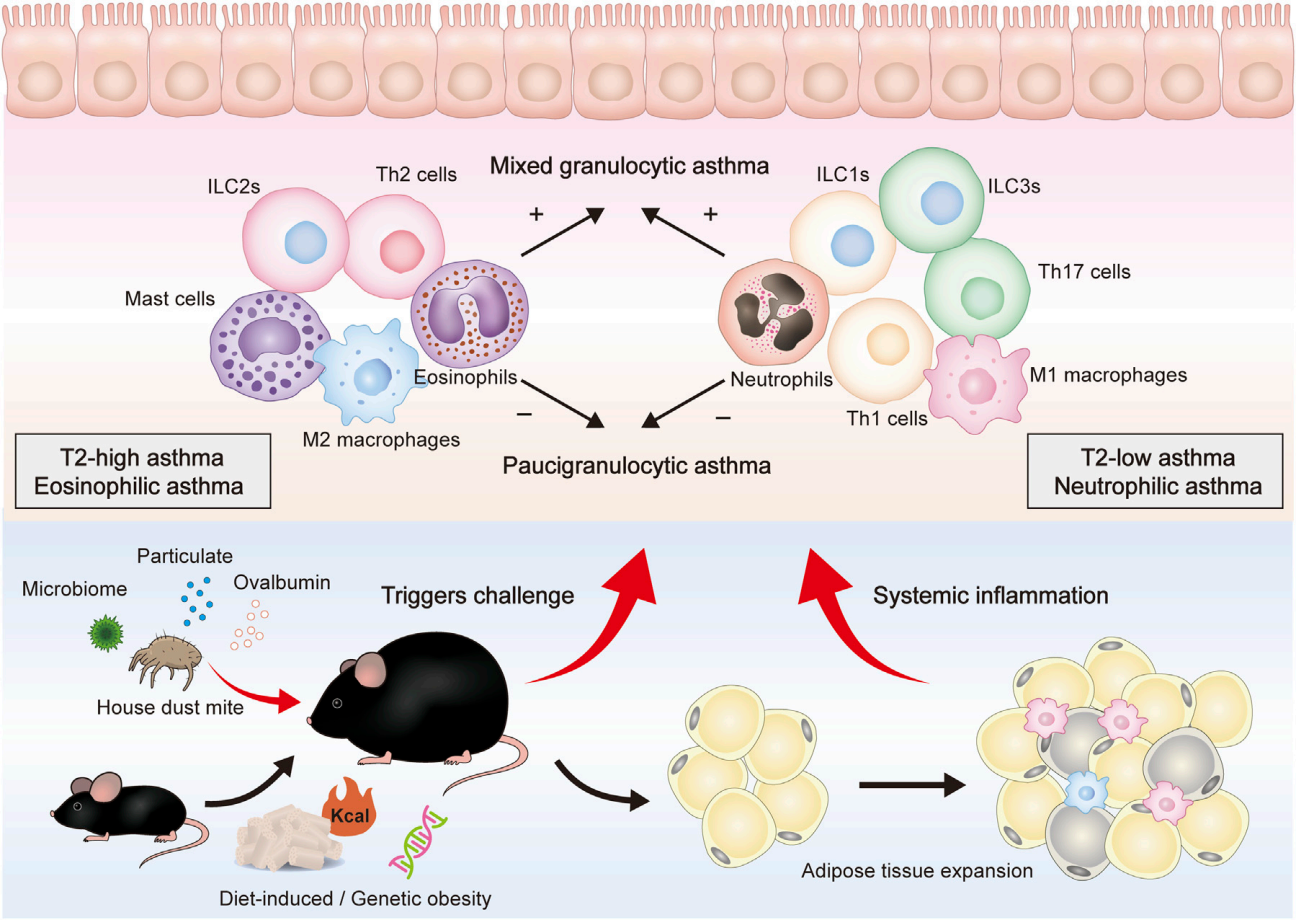
Overall presentation of obesity-related asthma development and the proposed role of different immune cells in the airway. Genes, diet, and other factors can lead to obesity in mice and are mainly characterized by adipose tissue expansion. After being stimulated by HDM, pollen, pollution, and other triggers, obese mice can further constitute an obesity-related asthma model. Obese asthma can be classified into two endotypes (T2-high, T2-low) or four phenotypes (eosinophilic, neutrophilic, mixed granulocytic, and paucigranulocytic). T2-low asthma is mainly composed of neutrophils, M1 macrophages, Th1 cells, Th17 cells, ILC1s, and ILC3s, whereas T2-high asthma is mainly composed of eosinophils, M2 macrophages, Th2 cells, and ILC2s. These cells can secrete cytokines and inflammatory mediators to induce epithelium damage and mucus secretion.

Neutrophils and eosinophils are the hallmark granulocytes of T2-low and T2-high asthma, respectively. Notably, four asthma phenotypes have been defined according to the cell counts of these two inflammatory cells in the sputum: eosinophilic asthma, neutrophilic asthma, mixed granulocytic asthma (MGA), and paucigranulocytic asthma (PGA) (Tliba and Panettieri, 2019). Sputum eosinophil and neutrophil counts are increased in MGA but have no change in PGA. These four phenotypes are observed in the lung tissue and bronchoalveolar lavage fluid (BALF) of asthmatic mice (Figure 1).

### Obesity-related asthma is a severe asthma phenotype

Obesity is a crucial risk factor for asthma exacerbation in both childhood and adulthood, while females are more likely to develop obesity-related asthma (Tashiro and Shore, 2019; Sansone et al., 2020; Halayko et al., 2021). Global Initiative for Asthma (GINA) 2022 lists asthma with obesity as a single phenotype, and this instruction indicates that obese asthmatic patients have prominent symptoms, corticosteroid resistance, and less eosinophilic airway inflammation (Global Initiative for Asthma, 2022). In obese asthmatics, impaired lung function and increased inflammatory cell infiltration are the main causes of severe symptoms (Peters et al., 2018; Miethe et al., 2020). Both of them leave obese asthmatic patients experiencing a heavy burden of uncontrollable symptoms and medication side effects.

Pulmonary physiology is significantly altered by the compression of adipose tissues in the obese population (Littleton and Tulaimat, 2017). The abnormalities of chest wall properties and respiratory muscle motion have profound effects on lung volume, gas transfer, and airway resistance, which are reflected in indicators, such as expiratory reserve volume, functional residual capacity, and residual volume (Littleton, 2012; Dixon and Peters, 2018; Huang et al., 2021). In addition to mechanical impediment, systemic inflammation contributes to pulmonary dysfunction in obesity. A significant correlation was observed between circulating obesity-related inflammatory molecules and forced expiratory volume in 1 s (FEV1), forced vital capacity (FVC), and FEV1/FVC ratio (Lecube et al., 2011; Brazzale et al., 2015). Hence, systemic inflammation is a critical modifier in controlling asthma symptoms and preventing asthma exacerbation.

Notably, the relationship between obesity and asthma appears to be bidirectional. Obese asthmatic patients exhibit worse asthma control and do not respond appropriately to standard asthma medications, in contrast to lean asthmatic patients. Patients even experience recurrence after receiving high-dose inhaled corticosteroid and β2-agonist treatment (Global Initiative for Asthma, 2022). Meanwhile, poorly controlled asthmatics usually do less strenuous exercise, an essential factor for obesity after suffering from asthma. Numerous studies have shown that sustained weight loss can improve asthma control and lung function (Freitas et al., 2018; Eslick et al., 2020). Diet changes, increased exercise, and bariatric surgery all effectively induce weight loss, which is related to the alleviation of asthma (Scott et al., 2013; Nyenhuis et al., 2018). Therefore, obesity is a significant risk factor for asthma development and needs to be assessed at all ages, and weight loss is helpful for asthma management.

## Low-grade chronic systemic inflammation in obesity

The adipose tissue is an energy-storage organ that maintains whole-body energy homeostasis. The dysfunctions of adipose tissues often cause systemic inflammation that affects multiple organs (Ouchi et al., 2011; Rohm et al., 2022). Another significant factor for obesity-related asthma is a skewed immune response: many adipokines and immune cells are altered in the obese state, resulting in systemic and airway inflammation (Bantulà et al., 2021).

### Asthma-related adipokines in obesity

Adipose tissues are distributed in several organs in the pathological process of obesity. In the obese state, adipokines, which include cytokines, chemokines, complement proteins, and other acute-phase molecules, contribute to the onset of obesity‐related comorbidities (Fasshauer and Blüher, 2015; Unamuno et al., 2018; Taylor, 2021). Adipokines are selectively altered in obesity and can cause pleiotropic effects, such as modulating angiogenesis, metabolism, and inflammation (Chang et al., 2020). Among the main adipokines, pro-inflammatory leptin and anti-inflammatory adiponectin play an important role in the development of obese asthma (Fasshauer and Blüher, 2015; Bantulà et al., 2021). Obese individuals have high levels of leptin, which is important in regulating satiety, appetite, and food intake (Zhao et al., 2021). Leptin levels in the blood positively correlate with adipose mass. Whereas serum levels of adiponectin decrease with obesity and are positively associated with insulin sensitivity (Fasshauer and Blüher, 2015; Unamuno et al., 2018). Other adipokines, such as IL-1β, IL-6, tumor necrosis factor-α (TNF-α), monocyte chemotactic protein (MCP-1), can also augment immune response in the distant airway under the trigger of aeroallergens (Shore, 2008; Recinella et al., 2020). In addition, adipokines can chemoattract immune cell recruitment, regulate immune cell polarization, and activate inflammatory signaling pathways.

### Asthma-related immune cells in obesity

Immune cells are crucial factors in the systemic inflammation of obesity. Innate and adaptive immune cells infiltrate adipose tissues at the onset of weight gain and act as major adipose tissue components to perpetuate an inflammatory state (Blüher, 2019). Notably, the frequency and count of asthma-associated immune cells are higher in the obese than in the lean state (Han and Levings, 2013; Chung et al., 2018). Immune cells linking asthma and obesity further affect the distant airway through pro-inflammatory function.

Macrophages can infiltrate adipose tissues and regulate the local metabolism (Sharma et al., 2018). The levels of M1 macrophages are more elevated in obese individuals than in lean individuals, and M1 macrophages residing in adipose tissue widely orchestrate local and systemic inflammatory responses. Circulating monocytes are recruited into adipose tissues by chemokines, such as MCP-1, and replace exhausted macrophages involved in inflammation. M1/M2 polarization is regulated by various immune cells in obesity-related systemic inflammation (Bai and Sun, 2015; Wensveen et al., 2015). In addition, AT neutrophils rapidly increase and produce effector proteins days after high-fat diet (HFD) administration (Talukdar et al., 2012). Circulating neutrophils are also activated in severely obese subjects (Brotfain et al., 2015). In contrast to neutrophils, eosinophils present anti-inflammatory functions in obesity, and their cell counts are lower in obese mice AT than in lean mice (Bolus et al., 2018). HFD-treated eosinophil-deficient mice show increased body fat and impaired glucose tolerance (Takeda et al., 2015). ILCs not only participate in innate immune defense against pathogens, but also act as a bridge between innate and adaptive immunity by orchestrating adaptive immune responses through direct contact or cytokine secretion (Everaere et al., 2018; Vivier et al., 2018). The ILC2s predominate in the lean white AT, contribute to AT homeostasis, and stimulate eosinophils and M2 macrophages by secreting IL-5 and IL-13 (Brestoff et al., 2015). By contrast, ILC1s can produce IFN-γ to drive M1 polarization and promote obesity-related inflammation (Sasaki et al., 2019). Increases in the levels of ILC3s are observed in adipose and lung tissues in obesity, and ILC3s can mediate the development of AHR in HFD-induced obese mice with NLRP3 and IL-1β (Kim et al., 2014).

Adaptive T and B lymphocytes play essential roles in regulating AT inflammation in obesity (McLaughlin et al., 2017; Misumi et al., 2019). Increased Th1 cells produce inflammatory molecules and induce M1 polarization in the development of obesity (Van Herck et al., 2019). By contrast, Th2 cells and their products reduce AT inflammation and facilitate the differentiation of M2 macrophages. Few Th2 cells are found in obese adipose tissues, and the number of Th2 cells is inversely associated with systemic inflammatory markers (Morin et al., 2017). In addition, an increase in Th17 cells is observed in the adipose tissues and spleens of mice with HFD, whose product IL-17 subsequently induces neutrophil recruitment and causes tissue damage (Endo et al., 2017). As pivotal cells conducting the humoral immune response, B lymphocytes regulate visceral adipose tissue inflammation by presenting antigens to T cells, secreting pro-inflammatory cytokines, and producing pathogenic antibodies IgG and IgA (DeFuria et al., 2013; Frasca et al., 2016; Luck et al., 2019). Hence, immune cells are extensively involved in obesity and associated systemic inflammation, and they are also potential key targets for the treatment of obese asthma.

## Establishment of obesity-related asthma mouse models

Owing to the increasing incidence of obesity-related asthma over the past few decades, identifying the underlying immunological mechanisms of this disease is imperative. However, mimicking the clinical characteristics of a human disease in mice remains a substantial challenge. Several obese asthma models have been established and used in investigating the complicated processes of fat accumulation and airway inflammation. A model of obesity-related asthma generally involves two main procedures: the establishment of obesity and the induction of experimental asthma.

### Mouse models of obesity

Establishing a successful obesity model is the first step in constructing an obesity-related model. Diet-induced obesity (DIO) is a standard and the most widely used method, in which mice are usually fed an HFD for several weeks (Kleinert et al., 2018). Some studies have combined a high-fat and high-sugar diet to induce obesity (Percopo et al., 2021). The C57BL/6 strain is mostly used in DIO models, whereas it is reported that BALB/c mice are resistant to developing obesity due to the protection from HFD (Kodela et al., 2018). A successful DIO model is defined as more than 20% body weight over the baseline (Vieira et al., 2020). However, a drawback is that a DIO model only displays a mild or moderate obesity. In contrast to this canonical model, genetically engineered mice are more susceptible to severe obesity, such as leptin-deficient (*ob/ob*) mice, leptin receptor-deficient (*db/db*) mice, and carboxypeptidase E-deficient (*Cpe* ^*fat*^) mice (Kleinert et al., 2018). Moreover, TALLYHO/Jng mice established for the model of non-insulin-dependent diabetes mellitus are used as a polygenic obese strain (Hunter et al., 2019). These genetic obese models have advantages in terms of mice’s age. Allergen stimulation can be given to genetically deficient mice at 6–8 weeks of age to construct an asthma model for comparison with wild-type or heterozygous mice (Shore, 2007). DIO mice do not have sufficient weight difference to induce an asthma model until at least 12–15 weeks of age. In *ob/ob* and HFD-induced obese mice suffering from airway diseases, IL-17A, NLRP3, and ILC3s are essential parts of inflammation, whereas type 2 responses may be unnecessary for an obese asthma phenotype (Kim et al., 2014; Mathews et al., 2014).

### Mouse models of asthma

Allergens are sensitized and challenged after an obese model has been established. Chicken egg ovalbumin (OVA) has been the most frequently used allergen due to its low cost and high purity. Mice are immunized *via* the peritoneal/subcutaneous route with OVA emulsified in aluminum hydroxide, a canonical Th2 response adjuvant (Khumalo et al., 2020; Azevedo et al., 2021). In ordinary asthma studies, two to three times of sensitization (OVA + adjuvant) and 1 week challenge (OVA) are used, and allergen challenges include inhalational, intranasal, and intratracheal administration (Dos Santos et al., 2018; Asayama et al., 2020). Although OVA-induced asthma is an intensive type 2 immune response and is widely used in obese asthma studies, only a few clinical asthma cases are caused by allogeneic proteins. House dust mite (HDM) -induced asthma is much more clinically relevant. HDM is generally sensitized and stimulated intranasally without adjuvants (Saglani et al., 2018; Bachus et al., 2019; Tibbitt et al., 2019). It is consistent with the clinical phenomenon that human asthma is mostly caused by the inhalation of natural allergens. HDM-induced asthma phenotypes include eosinophilic, neutrophilic, and mixed asthma (Tina Tan et al., 2019). Given the immune status of obesity, HDM is suitable for modeling obesity-related asthma. Other allergens, such as cockroaches, chitin, papain, and *Alternaria alternata*, are given *via* natural inhalation for the modeling of asthmatic mice. In addition, ozone (O_3_) and diesel exhaust particles (DEP) can be used to induce non-atopic asthma.

## Immune responses in the different obesity-related asthma mouse models

**FIGURE 2:**
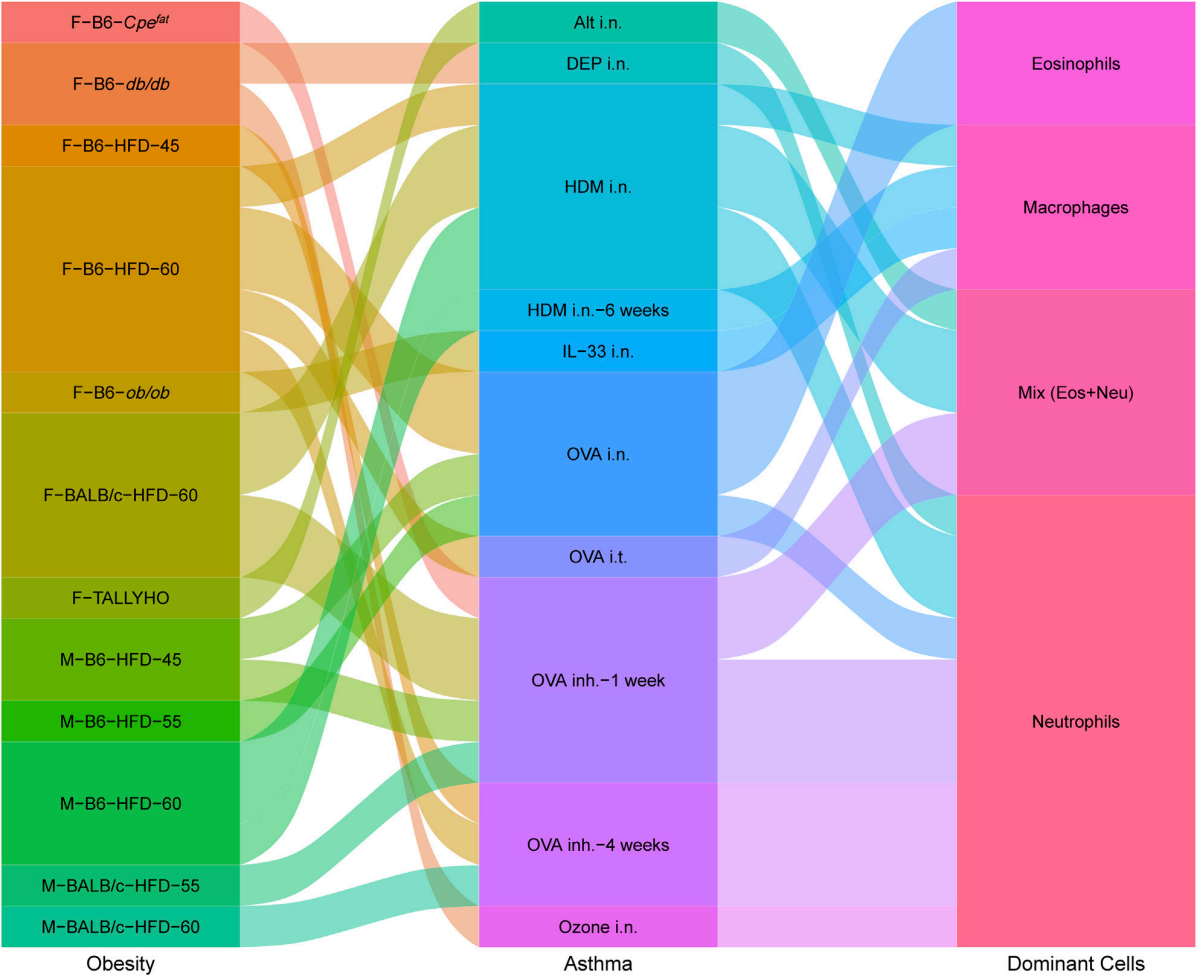
Interplay between obesity models, asthma models and dominant immune cells in obesity-related asthma. Obesity contains Sex–Strain–Obesity model-diet energy content; asthma contains allergens/triggers–administration methods–trigger time; dominant cells contain increased immune cells in lung tissues. The sex and strain of mice, obesity patterns, and allergen administration can impact inflammatory response. Airway immune responses are mainly dominated by neutrophils, eosinophils, macrophages, or a mix of neutrophils and eosinophils.

Changes in neutrophils and eosinophils demonstrate skewed immune responses and different asthma phenotypes. Various factors, such as strain, sex, age, diet, allergen, and exposure time, lead to distinct dominant cells (neutrophils, eosinophils, macrophages, and a mix of neutrophils and eosinophils) in airway inflammation (Figure 2). The modeling procedures and their impact on the immune responses will be discussed separately in the following.

### Female obesity-related asthma mouse models

As obesity-related asthma is more prevalent in female patients, using female mice to detect changes in immune response in obese asthmatics makes sense. Details of an obese asthma modeling protocol for female mice are shown in Table 1. Compared with non-obese asthmatic mice, obese asthmatic mice have more severe symptom profiles. In most studies, the proportion and count of neutrophils were significantly higher in the BALF and lung tissues of obese mice than those in lean asthmatics. IL-33 is commonly used as an activator of lung ILC2s in the study of allergic inflammation (Karagiannis et al., 2020). However, IL-33-treated *ob/ob* mice showed an increase in macrophages and a decrease in eosinophils (Kurokawa et al., 2021). Pulmonary ILC2s were increased in TALLYHO mice in response to *Alternaria alternata* extract (Toki et al., 2021). The dominant cells shifted from eosinophils to macrophages in the intratracheal OVA-challenged mice, in which the mobilization of eosinophils in response to allergens failed (de Vries et al., 2009; Kurokawa et al., 2021). In addition, HFD/HDM mice and *Cpe* ^*fat*^/OVA mice showed MGA phenotype related to ILC2s and ILC3s (Dahm et al., 2014; Everaere et al., 2016). Type 2-high and type 2-low airway inflammation were attenuated by nonantibiotic macrolide EM900 in the obese asthma model (Sadamatsu et al., 2020).^.^ Moreover, increases in eosinophils and type 2 cytokines were observed in the DEP-treated asthmatic lean mice (*db/+m*), but DEP induced skewed mild neutrophilic airway inflammation in obese (*db/db*) mice, and the effect was accompanied by elevated toxin and MCP-1 levels (Yanagisawa et al., 2014).

**TABLE 1:** Summary of the female obesity-related asthma mice and their immune response and findings.

Strain	Obesity Model	Start Age	HFD Time	Asthma Model	Trigger Time	Immune response	Finding	References
C57BL/6J	HFD (45%)	4–5	12	OVA inh.	4 weeks	BALF: Eos↓; Total Neu↑	Obesity-related asthma mice show higher oxidative stress and activation of NF-κB in lung tissue.	Liu et al. (2016), Liu et al. (2020)
C57BL/6J	HFD (60%)	4	15	OVA inh.	4 weeks	BALF: Total, Neu↑; Eos, Mac↓	Simvastatin treats obese asthma by improving dyslipidemia and decreasing leptin level.	Han et al. (2017)
C57BL/6	HFD (60%)	4	12	OVA i.n.	4 times	BALF: Total, Eos, Mac, IL-5↑; Serum: IL-17↑	Pravastatin treatment suppresses allergic airway infiltration and AHR in obese asthmatic mice by inhibiting Th2 and Th17, leptin, and p38 MAPK signaling pathways.	Lee et al. (2019)
C57BL/6	HFD (60%)	4	12	OVA i.n.	4 times	BALF: Total, Eos, Mac, IL-4, IL-5, IL-17↑	IL-17–leptin/adiponectin axis plays a key role in airway inflammation in obesity-related asthma.	Liang et al. (2018)
C57BL/6J	HFD (58%)	3–4	8–9	OVA i.t.	3 times	BALF: Mac↑; Eos, Lym, IL-5↓; Lung: Eos↓	High-fat dietary content redirects local immune responses to allergen in the lungs	de Vries et al. (2009)
C57BL/6J	HFD (42%)	4	12	OVA i.t.	4 times	BALF: Total, Neu, T cell↓; Lung: DC, T cell↓	Short‐term HFD feeding and associated metabolic alterations have protective effects in allergic asthma	Schröder et al. (2019)
C57BL/6J	HFD (60%)	4	10	HDM i.n.	5 times	BALF: Eos↑; Lung: IL-4, IL13, IL-17A, IL-33, IL-1β, ILC2s, ILC3s↑; Serum: IgE↑	HFD-induced obesity might exacerbate allergic airway inflammation through mechanisms involving ILC2s and ILC3s.	Everaere et al. (2016)
C57BL/6J	*ob/ob*	7–9	—	IL-33 i.n.	3 times	BALF: Mac↑; Eos, IL-5, IL-13↓	IL-33 with leptin induces airway inflammation and goblet cell metaplasia and enhances AHR.	Kurokawa et al. (2016)
C57BL/6J	*db/db*	6–8	—	Ozone i.n.	1 times (3 h)	BALF: Neu, IL-6, IL-17A, IL-23↑; Serum: Neu, IL-17A↑	IL-17A contributes to the augmented responses to ozone observed in db/db mice and IL-17A may mediate O_3_-induced AHR *via* gastrin-releasing peptide receptor	Shore et al. (2008), Mathews et al. (2018)
C57BL/6J	*db/db*	5	—	DEP i.n.	7 times	BALF: Lym Eos↓; Neu↑; Lung: IL-6, IL-1β, IL-5, IL-13, TNF-α↓	Obesity can affect susceptibility to DEP-induced airway inflammation, there is mild neutrophilic inflammation with attenuation of eosinophilic infiltration in the lungs DEP-treated db/db mice.	Yanagisawa et al. (2014)
C57BL/6J	*Cpe* ^*fat*^	4–9	—	OVA inh.	1 week	BALF: Total, Mac, Eos, Neu↑, IL-4, IL-13, IL-18↑; Serum: IL-18↑	OVA enhances airway obstruction in obese mice regardless of the genetic basis of obesity, whereas the OVA-induced pulmonary inflammation is dependent on the genetic modality of obesity induction.	Dahm et al. (2014)
TALLYHO	TALLYHO/JngJ	9–12	—	Alt i.n.	3 times	BALF: Neu, Lym, TSLP↑; Mac↓; Lung: IL-5, IL-13, ILC2s↑	GLP-1RA treatment inhibits aeroallergen-induced eosinophilic and neutrophilic airway inflammation in obesity-related asthma.	Toki et al. (2021)
BALB/c	HFD (60%)	5–6	10	OVA inh.	1 week	BALF: Eos↓ Mac↑; Serum: IgE↓; Lung: MC↑	Obesity affects allergic airway inflammation through mast cell influx and the release of TSLP and IL-25.	Silva et al. (2017)
BALB/c	HFD (60%)	5–7	14	OVA inh.	1 week	Lung: IL-4, IL-17A↑; Eos, IL-25, TSLP↓; Serum: IgE↓; IgG2a↑	Obesity affects the peripheral response to the allergen, promoting antibody production disturbances and the components of the germinal center.	Esteves de Oliveira et al. (2019)
BALB/c	HFD (60%)	6	11	HDM i.n.	4 times	BALF: Neu↑; Lung: IL-17A↑	Both type 2-high and type 2-low airway inflammation are attenuated by EM900 in obese asthma	Sadamatsu et al. (2020)
BALB/c	HFD (60%)	3–6	10	HDM i.n.	3 times	BALF: Total, Neu,↑; Lung: IL-17A↑	Saturated fatty acid augments HDM-induced neutrophilic airway inflammation in a HFD mouse model.	Tashiro et al. (2017)

Obesity model: types of obese mouse models and diet energy content; start age: age of mice used in studies after adaptive housing; HFD, time: duration of HFD, administration (weeks); asthma model: allergens/triggers and administration methods; trigger time: duration/times of allergens/triggers stimulation; immune response: changes in immune cells, cytokines, and antibodies in BALF, lung tissues, and blood. All upward or downward trends marked by arrows are compared with the lean asthma group; finding: main findings and conclusions of the study; HFD, high-fat diet; inh., inhalational administration; i.n., intranasal administration; i.t., intratracheal administration; DEP, diesel exhaust particles; OVA, Ovalbumin; HDM, House dust mite; Alt, *Alternaria alternata* extract; Der.p, Dermatophagoides pteronyssinus; BALF; Bronchoalveolar lavage fluid; Total, Total white blood cells; Eos, Eosinophils; Neu, Neutrophils; Mac, Macrophages; Lym, lymphocytes; DC, Dendritic cells; MC, Mast cells; ILC2s, Group 2 innate lymphoid cells; ILC3s, Group 3 innate lymphoid cells; TSLP, Thymic stromal lymphopoietin; ↑, increase; ↓, decrease.

Several biological processes and inflammatory signaling pathways were found in mouse models of obesity-related asthma. Notably, adipokines may serve as a link between obesity and asthma, such as leptin, TNF-α, and IL-6. Obese asthmatic mice have higher levels of leptin and an elevated leptin/adiponectin ratio in their sera, and the IL-17–leptin/adiponectin axis can boost airway inflammation (Han et al., 2017; Liang et al., 2018; Lee et al., 2019). Statins are the most commonly prescribed oral agents for lipid-lowering therapy, and statin-medicated obese patients display a lower degree of inflammation and lipid level than non-statin-medicated patients (Vieira-Silva et al., 2020). In addition, statins have pleiotropic anti-inflammatory and immune-modulating effects, demonstrating potential as effective treatments for obese asthma. Simvastatin can effectively improve airway inflammation and remodeling by regulating dyslipidemia and decreasing leptin levels (Han et al., 2017). Pravastatin treatment can suppress airway inflammation by inhibiting Th2 and Th17 responses and decreasing leptin expression and downstream p38 MAPK phosphorylation (Han et al., 2017; Lee et al., 2019). Moreover, the anti-obesity drug liraglutide, a glucagon-like peptide-1 (GLP-1) receptor agonist, can alleviate airway inflammation by suppressing NLRP3 inflammasome activity (Hur et al., 2021). GLP-1R agonists decrease Alt-Ext-induced airway neutrophilia in TALLYHO mice (Toki et al., 2021). The number of lung macrophages was higher in the HFD mice than in the standard chow mice. This change was related to an increase in the level of saturated fatty acids, which augment HDM-induced neutrophilic airway inflammation in obese asthmatic mice (Tashiro et al., 2017).

Since obesity, dominated by activation of neutrophils, M1 macrophages and Th17 cells, is a non-type 2 systemic inflammation, it is reasonable to assume that obese asthma mouse models are mostly T2-low asthma. Some studies have also found the infiltration of eosinophils and activation of Th2 cells and ILC2s in obese asthmatic mice. Regardless of which immune cells are elevated and dominant in the BALF or lung tissues, most studies have found that obesity can exacerbate airway inflammation (Schröder et al., 2019). However, one study indicated that short-term HFD feeding can attenuate the development of OVA-induced asthma. In the results, no change in Th2 differentiation was observed. Although no uniform standard for the duration of HFD has been established, defining a 12-weeks HFD as a short-term intervention is controversial. This protective effect of obesity on asthma may be explained by the “obesity paradox,” which states that obesity is associated with improved prognosis in people suffering from chronic diseases, such as COPD (Spelta et al., 2018).

### Male obesity-related asthma mouse models

The detailed modeling protocols of male obese asthma mice are shown in Table 2. In most studies, type 2 immune cytokines were associated with eosinophils, with the exception of one study in which eosinophils were reduced and macrophages were increased in BALF and lung tissue (Diaz et al., 2015). Similar to the female model, male obese asthmatic mice exhibited increased neutrophils and Th17 cells (Zhu et al., 2019). The inhibition of Notch signaling can suppress the Th17 response and alleviate AHR in obese asthmatic mice (Zeng et al., 2019). Notably, no changes in eosinophils and neutrophils were found in one study, only an increase in macrophages, and this effect was accompanied by decreased levels of IFN-γ, IL-5, IL-13, IL-6, and IL-17 in BALF (Ather et al., 2016). Other immune cells were identified in the male model. Increases in M1 and decreases in M2 and AMs were found in obesity-related asthma, possibly consistent with an obese state (Lee et al., 2021). NKT and CD69^+^NKT cells increased in the HFD/OVA-induced model. Synthetic alpha-galactose ceramide KRN7000 can significantly reduce airway inflammation by regulating cytokine secretion and intracellular calcium in NKT cells (Chen et al., 2021a).

**TABLE 2:** Summary of the female obesity-related asthma mice and their immune response and findings.

Strain	Obesity Model	Start Age	HFD Time	Asthma Model	Trigger Time	Immune response	Finding	References
C57BL/6J	HFD (60%)	6	15	OVA i.n.	5 times	BALF: Eos↓; Lung: Treg↓	AT inflammation in obesity exacerbates allergic inflammation by downregulating lung AdipoR1+ Tregs.	Ramos-Ramírez et al. (2020)
C57BL6/J	HFD (55%)	4	10	OVA i.n.	4 times (2 days)	BALF: Total, Eos, IL-6↓; IL-5, TNF-a, IL-10↑; Lung: Total, Eos↑	Obesity enhances eosinophil trafficking and delays their transit into lumen. Metformin inhibits TNF-α-induced inflammatory signaling and NF-κB-mediated iNOS expression in the lung of obese mice.	Calixto et al. (2010), Calixto et al. (2013)
C57BL/6	HFD (45%)	3–4	16	OVA i.n.	4 times	Lung: IL-17A, Th17↑; Serum: IL-1β, IL-6, IL-17A↑	Celastrol could suppress AHR through Th17 inhibition in obese asthmatic mice.Notch pathway is related to AHR and Th17 response in obese asthmatic mice	Zeng et al. (2018), Zeng et al. (2019)
C57BL6/J	HFD	3–4	20	OVA ihn.	4 weeks	Lung: NKT cell, CD69^+^NKT cell↑	KRN7000 ameliorates obese asthma by regulating NKT cytokine secretion and intracellular calcium	Chen et al. (2021a)
C57BL/6J	HFD (45%)	3–4	12	OVA ihn.	8 times	BALF: Eos↓; Neu, TNF-α↑; Serum: TNF-α↑	Adiponectin may protect against obesity-related asthma *via* activating the AMPK pathway	Zhu et al. (2019)
C57BL/6J	HFD (45%)	3–4	12	OVA ihn.	8 times	Lung: STAT3, STAT6↑	Leptin impacts the pulmonary inflammation of obese asthma by activating the STAT3 signaling pathway.	Chong et al. (2019)
C57BL/6J	HFD (45%)	—	25	HDM i.n.	5 times	BALF: Eos*↓; Lym*↑	Dysregulation of Pyruvate Kinase M2 promotes inflammation in obese asthmatic mice	Manuel et al. (2021)
C57BL/6	HFD (60%)	6–7	16	HDM i.n.	3times	Lung: iNOS↑	Altered NO metabolism in the obese-asthma contributes to steroid resistance.	Singh et al. (2018)
C57BL/6J	HFD (60%)	6	16	HDM i.n.	4 times	BALF: Mac*↑; Lung: IL-5, IL-13, IFN-γ, IL-6, IL-17A*↓	Diet-induced weight loss is effective in models of both inherent and allergic obese asthma. Structural, immunological, and microbiological factors contribute to the manifold presentations of obese asthma.	Ather et al. (2016)
C57BL/6J	HFD (60%)	16	—	HDM i.n.	4 times	BALF: Total, Eos, Neu, Lym↑; Lung: IL-13, IFN-γ, CD11b^+^DCs↑; Serum: IgE, IgG1↑	Obesity impairs the proliferation of DC-restricted progenitors *via* Adam17-p38 MAPK-dependent pathway, changes in DC precursors and DCs elicit an impaired immune response in allergic asthma.	Jaiswal et al. (2020)
C57BL/6	HFD (60%)	5	25	HDM i.n.	6 weeks	BALF: Eos*↓ Mac*, IL-2, IL-4, IL-5↑; Lung: Eos↓ Mac↑; Plasma: IgE*↑	HDM combined with obesity promotes mixed localized inflammatory responses and lack a predominance of Th2 biomarkers, which exhibit more severe and are less sensitive to dexamethasone regulation.	Diaz et al. (2015)
C57BL/6	HFD (45%)	4	10	Der.p i.n.	2 times	Serum: IgE↑; Lung: M1↑ M2↓ AMs↓	Specific lung microbiome abundance and metabolic signatures are related to obese allergic asthma	Lee et al. (2021)
C57BL6/J#	HFD (60%)	3–4	16+	Ozone i.n.	1 times	BALF: IL-6, sTNFR1, sTNFR2↑	Innate AHR and enhanced O3-induced pulmonary responses are consistent features of obese mice.	Johnston et al. (2008)
C57BL6/J*#*	*ob/ob*	15–18	—	OVA i.n.	6 times	BALF: TNF-α IL-10↑ IL-6↓	OVA challenge in *ob/ob* mice promotes lung Eos accumulation, and delay their transit to airways lumen.	Lintomen et al. (2012)
BALB/c	HFD (55%)	3–4	8	OVA ihn.	1 week 8 times	BALF: Total, Neu, IL-4, IL-6, TNF-α, IL-1β IL-17, IL-18↑ IL-10↓; Lung: IL-1β, NLRP3↑	Serum vitamin D levels is associated with obese asthma, and NLRP3 inflammasome may play a role in this disorder.	Zhang et al. (2017)
BALB/c	HFD (60%)	3–4	14	OVA ihn.	4 weeks	BALF: Eos↓; IL-1β, IL-17A↑; Serum: IL-1β↑	LGWWJX protects obesity-related asthma through mechanisms including the inhibition of the IL-1β/ILC3/IL-17A/AHR axis, anti-inflammatory effects, weight loss, and the regulation of lipid metabolism.	Ma et al. (2021)

Obesity model: types of obese mouse models and diet energy content; start age: age of mice used in studies after adaptive housing; HFD, time: duration of HFD, administration (weeks); asthma model: allergens/triggers and administration methods; trigger time: duration/times of allergens/triggers stimulation; immune response: changes in immune cells, cytokines, and antibodies in BALF, lung tissues, and blood. All upward or downward trends marked by arrows are compared with the lean asthma group; finding: main findings and conclusions of the study; HFD, high-fat diet; inh., inhalational administration; i.n., intranasal administration; i.t., intratracheal administration; DEP, diesel exhaust particles; OVA, Ovalbumin; HDM, House dust mite; Alt, Alternaria alternata extract; Der.p, Dermatophagoides pteronyssinus; BALF; Bronchoalveolar lavage fluid; Total, Total white blood cells; Eos, Eosinophils; Neu, Neutrophils; Mac, Macrophages; Lym, lymphocytes; DC, Dendritic cells; MC, Mast cells; TSLP, Thymic stromal lymphopoietin; sTNFR, soluble Tumor necrosis factor receptor; LGWWJX, Linggan Wuwei Jiangxin formula; ↑, increase; ↓, decrease; *, no significant difference; #, results are pooled from male and female mice.

Metformin, which treats obesity comorbidity diabetes, benefits patients with asthma or obesity (Sanchez-Rangel and Inzucchi, 2017). A study on male mice has shown that metformin can attenuate obesity-related asthma by inhibiting the TNF-α-induced inflammatory signaling and NF-κB-mediated iNOS expression in lung tissues (Calixto et al., 2013). However, metformin did not affect ozone-induced inflammation in female *db/db* mice (Shore et al., 2008). Changes in leptin in males were similar to the shift in females (Chong et al., 2019). Many studies have demonstrated that leptin promotes pulmonary inflammation by activating the STAT3 signaling pathway (Zhu et al., 2019). By contrast, adiponectin exerted protective effects due to its anti-inflammatory and anti-oxidative functions. NLRP3 inflammasome, caspase-1, and IL-1β increased in female obese asthmatic mice, and this effect was also observed in male obese asthmatic mice and was associated with serum vitamin D levels (Zhang et al., 2017). In addition, obese asthma mice exhibited more severe symptoms that were less sensitive to dexamethasone regulation than lean asthmatics. The feature of steroid resistance was related to mixed localized inflammatory responses, shifted cellular infiltration, and lack of Th2 biomarkers (Diaz et al., 2015). The abundance of a specific lung microbiome differed between lean and obese mice exposed to *Der. p*, which revealed metabolic signatures related to an obese asthma phenotype (Lee et al., 2021). Traditional Chinese medicine is effective in treating obese asthma, such as celastrol, which can suppress AHR through Th17 inhibition (Zeng et al., 2018). Linggan Wuwei Jiangxin formula (LGWWJX) can protect against obese asthma most likely through mechanisms, including the inhibition of the IL-1β/ILC3/IL-17A/AHR axis and the regulation of lipid metabolism (Ma et al., 2021).

### Offspring asthma in maternal obese mice

Increasing evidence suggests that maternal obesity is a primary determinant of offspring health during childhood and adulthood (Godfrey et al., 2017). Research has indicated that a high pre-pregnancy BMI and excessive gestational weight gain could influence the risk of asthma and atopic disease in offspring (Harpsøe et al., 2013; Dumas et al., 2016). Meta-analyses have shown that maternal obesity and weight gain during pregnancy increase asthma risk in children and polarize immune response toward Th1 rather than Th2 (Forno et al., 2014; Nyambuya et al., 2020). Numerous studies have found that maternal HFD can affect the lung and somatic growth of the offspring. The lung volume and morphology of the offspring were affected by *in utero* exposure to maternal inflammation, which was mainly induced by metabolic dysfunction under maternal HFD and independent of inflammation in individual offspring (Smoothy et al., 2019). Other studies have indicated that maternal HFD can alter offspring’s plasma and hepatic fatty acid composition, secrete inflammatory markers, and modify lung development. Maternal diet composition may be more important than maternal obesity (Losol et al., 2021). During lactation, maternal HFD can cause offspring to gain weight. Early-onset obesity further dysregulated the pulmonary adipokine/insulin signaling pathway, leading to an asthma-like disease in adulthood (Dinger et al., 2016). Hence, maternal obesity is an effective way to recapitulate the clinical phenotypes of pediatric obesity or pediatric obese asthma.

Although maternal obesity can markedly influence the incidence of asthma in offspring, modeling procedures are more complicated than adult models. Immune response in early-onset obese mice exposed to allergens has not been fully explored. HFD was administered before mating and kept throughout pregnancy and lactation in a model of pediatric obese asthma. Then, offspring were sensitized to OVA at 6 weeks of age (E-Lacerda et al., 2019). The results showed that the progeny of the obese mice displayed exacerbated responses and intense lung remodeling in the OVA-induced asthma model, which are featured by elevated IL-4, IL-13, TNF-α, TGF-β levels, leukocyte infiltration and collagen deposition. Therefore, maintaining health during pregnancy and lactation may be an effective intervention for pediatric asthma. Mouse models of pediatric obesity-related asthma need more attention and exploration.

### Skewed immune response in different obesity-related asthma mouse models

Obese asthma is markedly heterogeneous, and its pathogenic state is not a single type of immune response. Previous studies have discussed mouse models combining obesity and asthma and have shown much interest in the effect of obesity on allergic and non-allergic obese asthma (Suratt, 2016; Chen et al., 2021b). In the murine model, non-allergic obese asthma was defined as airway inflammation in an obesity model without allergy stimulation. Such models are more similar to spontaneous airway hyperreactivity in obesity. The allergic innate immune response to HDM or papain exposure was discussed, whereas OVA exposure was related to an allergic adaptive immune response (Chen et al., 2021b). However, allergy and non-allergy cannot be distinguished simply by the presence or absence of triggers, nor should the distinction between innate and adaptive immune responses be classified according to allergen type.

**FIGURE 3:**
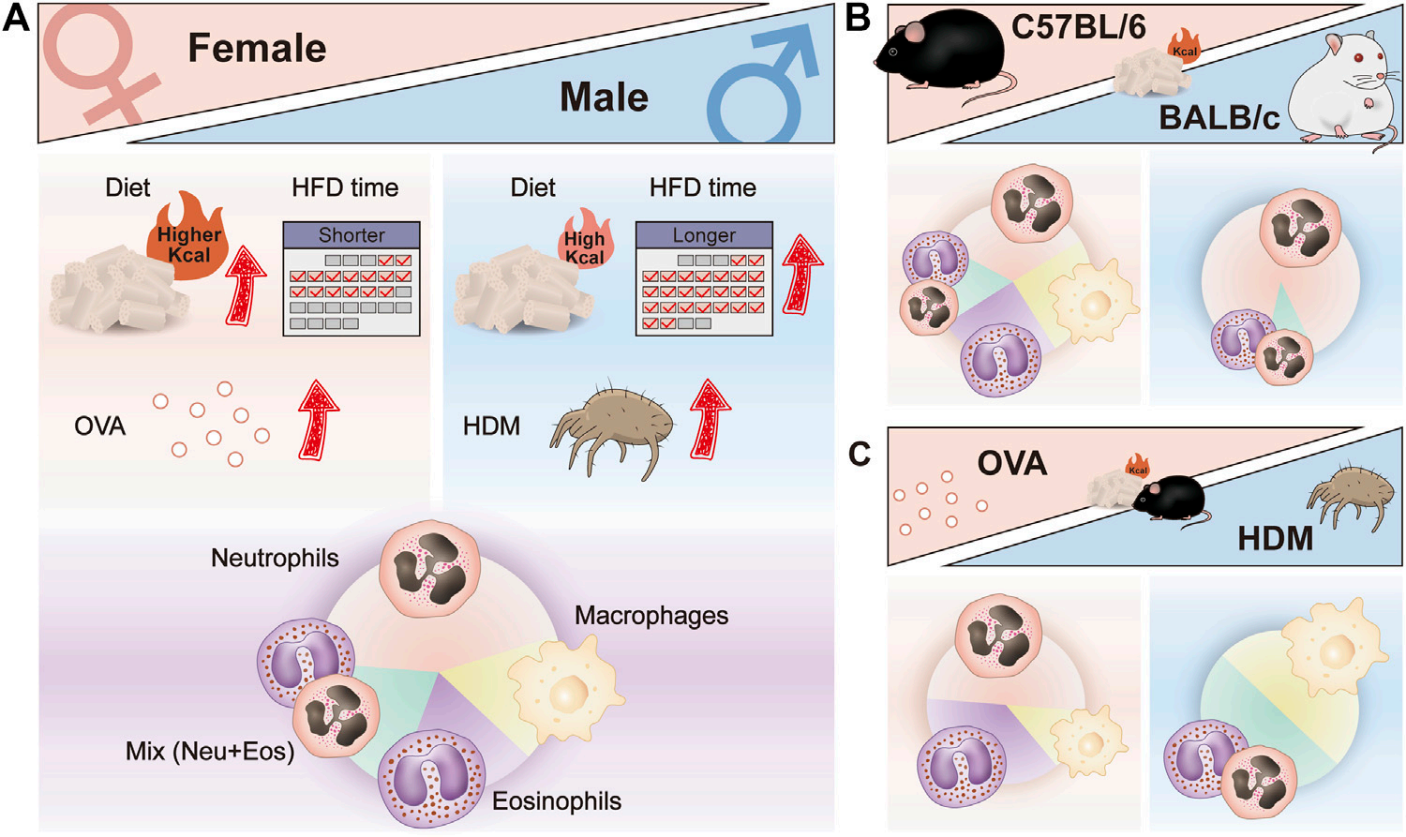
Skewed immune responses in different mouse models of obesity-related asthma. **(A)** Different factors in the construction of female and male mouse models of obesity-related asthma; different dominant immune cells in the airway of female and male mouse models of obesity-related asthma. **(B)** Different dominant immune cells in the airway of HFD-induced C57BL/6 and BALB/c mouse models of obesity-related asthma. **(C)** Different dominant immune cells in the airway of HFD/OVA and HFD/HDM induced C57BL/6 mouse models of obesity-related asthma. HFD, high-fat diet; OVA, Ovalbumin; HDM, House dust mite.

According to the inflammation profiles in obese asthmatic mice described above, we can conclude that modeling treatment results in a biased immune response with dominant neutrophils, eosinophils, macrophages, and a mix of neutrophils, and eosinophils (Figure 2). No significant changes in neutrophils and eosinophils were observed in the airways of macrophage-dominated obese asthmatic mice. Changes in macrophages may be related to macrophage activation and infiltration in the obese state. Further research is needed in the future to clarify the subsets of macrophages and confirm whether macrophage-dominated asthma can be equated to the PGA phenotype. In addition, skewed immune responses are observed in different mouse models of obesity-related asthma (Figure 3A). Although females have a heightened propensity to develop obesity-related asthma, this disease is not an exclusively female disease. Female mice were usually given a higher calorie diet than males, and males had a more extended period of diet-induced obesity than females. Moreover, most female mice used OVA as an allergen in the asthma model section, while only about half of the males used OVA (the others used HDM). In terms of immune response and dominant cells, both females and males were predominantly neutrophilic in obese asthma.

Grouping in different ways can reveal other factors that influence the response, such as strain and allergy type. Most studies used C57BL/6 mice for modeling, and only a few experiments utilized the BALB/c strain. When comparing the immune responses of different strains that were all provided with HFD, the airway inflammation in BALB/c mice was almost entirely neutrophils infiltrated (Figure 3B). In addition, in HFD-treated C57BL/6 mice, the type of allergen had an effect on the immune response, with predominantly eosinophils or neutrophils in the airways of HFD/OVA-treated mice and predominantly macrophages and a mix of neutrophils and eosinophils in HFD/HDM-treated mice (Figure 3C). Moreover, the mode of allergen administration, the frequency and duration of sensitization and stimulation may also affect the airway immune response in obese asthmatic mice.

### Prospects for the improvement of obesity-related asthma mouse models

The critical goal of obesity-related asthma models is to mimic essential clinical characteristics and investigate the immune mechanisms involved. The model of obesity-related asthma is similar to the superimposed model of obesity and asthma. The obesity evaluation system of the mouse model is limited, most studies use a 20% difference in body weight in evaluating obesity and allowing subsequent asthma induction. In clinical practice, obesity can be measured by BMI, waist circumference, waist-to-hip ratio, abdominal height, and skinfold thickness. Hence, weight differences may not be the most accurate indicator of fat accumulation. More comprehensive and effective obesity assessment systems are needed for selecting the right time for allergen interventions, which will improve the success rate of the obese asthma model. In addition, the type of allergen is limited in an obesity-related asthma model. For a long time, there is a great distance between animal experiments and clinical practice in the evaluation system of asthma models. The clinical diagnosis of asthma is mainly based on medical history, physical signs, and lung function tests. Histopathological sections and increased inflammatory cells in BALF as the criteria for successful models are rather one-sided.

Common pathogens, such as pollen, cockroaches, papain, fungi, and even viruses, can promote diverse immune responses in lung tissues without adjuvants, providing additional options for future models (Niespodziana et al., 2020). Given that animal models need to be clinically oriented, natural obesity factors (such as HFD) and natural allergens (such as HDM) are the best options. However, the genetic and maternal obese model can be used in detecting childhood immune response due to the high age of DIO mice. Mimicking critical clinical features in animal models and enriching the evaluation system of obesity, asthma, and obesity-related asthma models, these factors can facilitate an in-depth understanding of the immune mechanisms that contribute to obesity-related asthma.

## Conclusion

Obesity-related asthma is considered a severe asthma phenotype that presents tremendous challenges for clinical treatment. As a result of excessive energy intake, adipose expansion, and metabolic dysfunction, obesity can cause chronic low-grade inflammation in the body. Systemic inflammation in obesity is a critical risk factor for asthma development and severe symptoms. Although different types of immune responses have been observed in basic and clinical studies of obesity-related asthma, the molecular mechanisms and mediators involved are not yet clear. In addition to enabling detailed pathogenesis studies, a suitable model will also provide appropriate diagnostic criteria and therapeutic advice. This review summarizes the different approaches for establishing mouse models of obesity-related asthma and discusses the airway immune responses in different models. Many factors can affect the success of modeling and bias in immune response, such as the sex, strain, age of study subjects, dietary energy content, allergen type, and the timing and mode of diet/allergen administration. Trends in the dominant immune cells vary across studies, including neutrophils, eosinophils, macrophages, and a mix of neutrophils and eosinophils. Our knowledge regarding disease progression in obesity-related asthma, such as model assessment systems, and the subsets and functions of immune cells have many considerable gaps. Future studies are warranted to obtain a stable obese asthma model that fits the clinical profile and immune response, and this paper can provide some support for subsequent studies.
